# Access to anticancer and orphan medicines through compassionate use programs and named patient basis in seven European countries

**DOI:** 10.1016/j.esmoop.2025.105855

**Published:** 2025-11-13

**Authors:** N. Rosenberg, H.C. Post, T. Schutte, S.J. de Visser, I. Bartelink, A.M.G. Pasmooij, H.W.M. van Laarhoven, C.E.M. Hollak

**Affiliations:** 1 Medicine for Society, Platform at Amsterdam UMC – University of Amsterdam, Amsterdam, The Netherlands; 2 Department of Endocrinology and Metabolism, Amsterdam UMC, Amsterdam Gastroenterology Endocrinology Metabolism (AGEM) Research Institute, Expertise Centre for Inborn Errors of Metabolism, MetabERN, University of Amsterdam, Amsterdam, The Netherlands; 3 Department of Medical Oncology, Amsterdam UMC, Location University of Amsterdam, Amsterdam, The Netherlands; 4 Cancer Center Amsterdam, Cancer Treatment and Quality of Life, Amsterdam, The Netherlands; 5 Department of Medical Oncology, The Netherlands Cancer Institute (NKI-AVL), Amsterdam, The Netherlands; 6 Centre for Future Affordable & Sustainable Therapy Development (FAST), Leiden, The Netherlands; 7 Department of Clinical Pharmacy & Pharmacology, Amsterdam UMC, Location University of Amsterdam, Amsterdam, The Netherlands; 8 Dutch Medicines Evaluation Board, Utrecht, The Netherlands; 9 Division of Pharmacoepidemiology and Clinical Pharmacology, Utrecht Institute for Pharmaceutical Sciences, Utrecht University, Utrecht, The Netherlands

**Keywords:** early access, pre-authorization access, compassionate use, named patient, orphan medicines, anticancer medicines

## Abstract

**Background:**

In the European Union (EU), anticancer and orphan medicines are often granted marketing authorization based on surrogate endpoints and limited clinical trial data. Driven by unmet medical needs and the urgency of providing access beyond clinical trials, there is growing interest in early access, such as compassionate use programs (CUPs) and named patient basis (NPB). Additionally, limited clinical evidence can hinder health technology assessments and, when combined with high costs, delay reimbursement negotiations and patient access. Hence, it is crucial to explore CUPs and NPBs including pricing and reimbursement aspects.

**Design:**

This study includes a policy analysis to evaluate CUPs and NPBs in seven high-income European countries (Belgium, France, Germany, Netherlands, Norway, Switzerland, and the UK). We collected data on regulatory characteristics, including reimbursement aspects, from national health authority resources and direct consultations. In an in-depth examination, we assessed CUPs of anticancer and orphan medicines authorized in 2021 and 2022, focusing on availability, duration, and geographic distribution.

**Results:**

Our analysis reveals variability in national regulations, with inconsistent reimbursement options for CUPs and NPBs. For NPBs, reimbursement was often unregulated. The in-depth examination of CUPs revealed disparities in availability and duration before and after EU marketing authorization. We identified 36 CUPs, with 3-9 CUPs per country. Each CUP was available in up to four countries.

**Conclusion:**

We recommend minimizing disparities between CUPs and NPBs across Europe to ensure equitable access for patients with high unmet medical needs. Reducing these differences is essential to protect patients from feeling compelled to travel abroad or bear the financial burden of obtaining medicines that are not authorized in their home country.

## Highlights

• On a national level, CUPs are more tightly regulated than NPBs, but characteristics vary across countries.
• Compared with NPBs, CUPs are more likely to have regulations ensuring that patients do not bear the costs.
• Examination of CUPs for orphan and anticancer medicines demonstrated differences in both availability and duration.

## Introduction

Novel medicines, particularly anticancer and orphan medicines, are frequently authorized in the European Union (EU) based on surrogate or intermediate endpoints, such as biomarker response or progression-free survival.^1^^,^^2^ This approach is driven by the need for personalized cancer treatments and the focus on molecular subtyping of cancer types, which fosters the development of tailored therapies, but limits the options for large-scale randomized trials. Despite the limited clinical evidence, a positive opinion by the Committee for Medicinal Products for Human Use (CHMP) may be formed when the benefit–risk balance is considered favorable, based on promising effects on early endpoints, expectations of long-term efficacy, and an acceptable safety profile.^1^ Moreover, in the context of orphan medicines, conducting large clinical trials that convincingly demonstrate effects on clinically relevant outcomes is challenging, particularly for rare diseases with a slowly progressive course. Reflecting efforts to address various types of uncertainties, a positive CHMP opinion can lead to standard marketing authorization (MA), conditional MA, or MA under exceptional circumstances.^1^^,^^3^ Notably, 90% of medicines authorized through pathways such as conditional MA relied on surrogate endpoints in their pivotal trials, yet lacked studies that validate these endpoints.^1^

Many cancer (sub)types and rare diseases still lack authorized treatments, despite the development of numerous innovative therapies, illustrated by the nearly 3000 unauthorized medicines granted orphan designation.^4^ Given the unmet medical needs of patients and high expectations surrounding these promising yet unauthorized medicines, there is an urgent call for earlier access to effective and safe medicines. Simultaneously, we—as a society—face a social responsibility to ensure that medicines remain accessible for patients at a reasonable price.

Following MA, national health technology assessment (HTA) bodies that evaluate (cost-)effectiveness of new treatments may be hindered by limited data on clinical effectiveness and lack of comparators.^5^^,^^6^ Moreover, high prices exceeding national reimbursement thresholds and uncertainties in clinical outcomes may conflict with the willingness-to-pay. These factors might prolong HTA processes and contribute to extended reimbursement negotiations between payers and MA holders (MAHs), potentially delaying patient access while awaiting reimbursement decisions, which—even if positive—can take >1 year.^1^^,^^5^, ^6^, ^7^

There is European Commission (EC) legislation that provides provisions for accessing medicines before MA.^8^, ^9^, ^10^ These provisions include compassionate use programs (CUPs) for groups of patients and named patient basis (NPB) for individuals (see Box 1 for legal definition). CUPs allow for the use of unauthorized medicines currently studied in clinical trials or being evaluated by the European Medicines Agency (EMA), typically for a cohort of patients with chronically debilitating and/or life threatening diseases.^11^ CUPs may extend after MA, pending HTA and reimbursement decisions (Supplementary Figure S1).^12^ This ensures that, during this period, patients can access authorized yet non-reimbursed medicines. NPBs are designed for individual patients, under the responsibility of the prescribing physician.^13^ NPBs can serve multiple purposes, including facilitating access before MA and enabling the import of medicines authorized in other countries. The implementations of CUPs and NPBs can vary due to national law and practice, reflecting the flexibility of individual EU member states.^14^ Besides, the acceptance of charging costs by manufacturers for pre-authorization medicines can vary across the EU, depending on national laws and practices.^15^

Box 1 **Legal definition compassionate use programs and named patient basis according to European legislation**

Compassionate use program [art 83. Regulation (EC) No 726/2004]^11^: ‘compassionate use’ shall mean making a medicinal product (…) available for compassionate reasons to ‘a group of patients with a chronically or seriously debilitating disease’ or whose disease is considered to be ‘life-threatening’, and who ‘cannot be treated satisfactorily by an authorized medicinal product’. The medicinal product concerned must either be the ‘subject of an application for a marketing authorization’ (…) or must be ‘undergoing clinical trials’. Named Patient Basis [art 5. Directive 2001/83/(EC):]^12^: A Member State may, in accordance with legislation in force and ‘to fulfill special needs’, exclude from the provisions of this Directive medicinal products supplied in response to a ‘bona fide unsolicited order, formulated in accordance with the specifications’ of an ‘authorized health-care professional’ and for ‘use by an individual patient’ under his ‘direct personal responsibility’.

The varying regulations for CUPs and NPBs across Europe contribute to a heterogeneous and complex landscape.^16^^,^^17^ With the ultimate goal of creating harmonized access routes, this paper aims to evaluate both CUPs and NPBs by focusing on regulatory characteristics as well as the associated costs and reimbursement aspects in seven high-income European countries. Additionally, this study seeks to assess the availability of medicines through CUPs in these countries.

## Materials and methods

### Regulatory characteristics of CUPs and NPBs

We conducted a policy analysis to explore the regulatory characteristics of CUPs and NPBs in high-income European countries. Regulatory characteristics comprised general information and data on charging costs and reimbursement aspects that were collected from publicly available online resources from national health authorities (Table 1, Supplementary Table S1, available at https://doi.org/10.1016/j.esmoop.2025.105855):

• Name of the route
• Responsible authority
• Applicant
• Duration
• Possibilities for extension
• Duration of assessment by the regulatory authority
• Level of access (e.g. national, regional, hospital)
• Additional approval requirements
• Application fee
• National reimbursement possibilities
• Reimbursement organization
• Applicant for reimbursement
• Payer
• Permissibility of charging the patient
• Other costs involved (i.e. hospitalization or out-patient stay)

**Table 1:** Description of regulatory characteristics

Regulatory characteristic	Description
Name of the pre-authorization access route	The official name of CUP or NPB route at a national level
Responsible authority	The authority responsible for the authorization of the program at a national level
Applicant	The entity that has to apply for the pre-authorization access route to the responsible authority
Duration	The standard duration of the pre-authorization access route
Possibilities for extension	Whether the pre-authorization access route can be extended or not
Duration of assessment by the responsible authority	Time indicated by the responsible authority to assess the application of the pre-authorization access route
Level of pre-authorization access	The administrative level at which pre-authorization access can be granted (e.g. national, regional, hospital level)
Additional requirements	Any other requirements for pre-authorization access routes
Application fee	Whether a fee is associated with the application to the responsible authority
National reimbursement possibilities	Availability of reimbursement possibilities for pre-authorization access routes at the national level
Reimbursement organization	The national organization that is responsible for reimbursement of pre-authorization access routes
Applicant for reimbursement	The entity that has to apply for reimbursement of pre-authorization access program
Payer	The entity that is responsible for paying the medicinal product in the pre-authorization access program
Permissibility charging patient	Whether the patient can be charged for the medicine in the pre-authorization access program or not
Other costs involved	Additional costs such as hospitalization or out-patient stay

CUP, compassionate use program; NPB, named patient basis.

A sample was selected based on prior research on time to reimbursement following MA for the EU (EU-MA)^7^ in high-income West European countries with publicly available (reimbursement) data to ensure meaningful comparison. This enables an overview and comparison of pre- and post-authorization access in seven high-income West European countries. This included five European Economic Area (EEA) countries (Belgium, France, Germany, the Netherlands, and Norway), and two non-EEA countries (Switzerland and the UK).

Data on regulatory characteristics were collected between June and September 2023 with a reciprocal review process. Any discrepancies were resolved through double-checking and discussion. In case data could not be collected through publicly available online resources, experts from relevant national health authorities were consulted. These experts were identified through snowball sampling, starting with initial contacts from our regulatory network, who referred us to national experts in the selected countries. Personal information requests were subsequently sent via email. In total, 12 experts from seven countries were contacted for information related to CUPs and NPBs. If the requested information was still unavailable after the personal expert consultation, it was classified as unspecified. Regulatory characteristics were compared across countries to identify variations and hurdles.

### In-depth examination of CUPs for anticancer and orphan medicines

In addition to the analysis of regulatory characteristics, we conducted an in-depth examination of CUPs for anticancer and orphan medicines authorized in 2021 and 2022. Due to the absence of publicly listed NPBs, our focus was solely on CUPs. EU-authorized medicines were extracted from the European public assessment report (EPAR) database (retrieved July 2023).^18^ The data were verified and supplemented with data from the European Medicines Regulatory Database (retrieved February 2025). The data comprised the trade name, active substance, indication, MAH, EU-MA date, orphan medicine status, accelerated assessment, conditional MA, MA under exceptional circumstances, and priority medicine (PRIME) status (Supplementary Table S1, available at https://doi.org/10.1016/j.esmoop.2025.105855). Only originator anticancer and orphan medicines authorized in 2021 and 2022 were included.

To gather information on CUPs for these medicines, we consulted various sources (Supplementary Table S2, available at https://doi.org/10.1016/j.esmoop.2025.105855), including websites of national competent authorities (NCAs) and, where applicable, reimbursement organizations (Supplementary Table S3, available at https://doi.org/10.1016/j.esmoop.2025.105855). Switzerland was excluded from this analysis due to the lack of publicly available CUP end dates. Authorized medicines and CUPs were matched by cross-checking tradenames and indications. The data gathered focused on the start and end dates of CUPs according to the NCA. Data collection was conducted by authors NR and HCP between September 2023 and February 2025 to ensure the completion of CUPs. Any discrepancies were resolved through verification and discussion.

The start and end dates were used to calculate the duration of each CUP and compare these dates to the EU-MA date. Descriptive statistics were applied to summarize the findings. Additionally, we analyzed the number of CUPs available per country and the number of countries where each CUP was available.

## Results

### Regulatory characteristics of CUPs

The regulatory characteristics of CUPs across the seven countries revealed both similarities and differences. All countries have established regulatory pathways for CUPs, overseen by their respective NCAs (Table 2), which required manufacturers to submit applications to that national authority. In Belgium, in addition to manufacturers, the Minister of Social Affairs held the authority to submit applications to the NCA.^20^ In three countries—Belgium, Switzerland, and the UK—manufacturers were required to pay a fixed fee per CUP application submitted to the NCAs.^19^^,^^31^^,^^33^

**Table 2:** Overview of routing for compassionate use programs in seven European countries

Country	Program name	Responsible authority	Applicant	Duration	Extension possible	Assessment period	Level	Additional approval requirements	Fee to the authority
Belgium	Compassionate use program^19^	FAGG^19^	Minister of Social Affairs, manufacturer^20^	Until commercially available^21^	N/A	55 Working days excluding clockstop(s)^22^	National^19^	Only applicable to non-authorized medicines (any indication)^19^ [A Schiel (personal communication)]	Yes^19^
	Unmet medical need program^19^	FAGG^19^	Minister of Social Affairs, manufacturer^20^	Until reimbursed for the indication in the unmet medical need program [A Schiel (personal communication)]	N/A	55 Working days excluding clockstop^22^	National^19^	Only applicable to authorized medicines that are not yet authorized or reimbursed in another indication [A Schiel (personal communication)]	Yes^19^
France	Autorisation d'accès précoce^23^	ANSM and HAS^23^	Manufacturer^24^	1 Year^23^	Yes^23^	Approximately 60 days^23^	National^23^	N/A	No^23^
Germany	Arzneimittel-Härtefall-Verordnung^25^	BfArM and PEI^26^	Responsible person of the manufacturer^25^	1 Year (unless: —if the responsible person terminates it prematurely,—if the competent higher federal authority raises objections against its continuation or^25^—if the medicinal product has been licensed and is in fact available on the market)	N/A	2 Weeks (longer periods apply for genetically modified organisms)^25^	National^26^	N/A	No^25^^,^^26^ [A Berger (personal communication)]
Netherlands	Compassionate use program^27^	CBG. Program is supervised by the inspectorate IGJ^27^	Manufacturer^27^	1 Year^27^	Yes^27^	6 Weeks^28^	National^27^	N/A	No^27^
Norway	Compassionate use program^29^	DMP-NoMA^29^	Manufacturer^29^	1 Year^29^	Yes,^29^ until commercially available [A Schiel (personal communication)]	35 Days^29^	National^29^	Hospital management has to approve^29^	No^29^
Switzerland	Temporary authorization of use of an unauthorized medicinal product^30^	Swissmedic^30^	Manufacturer^31^	Temporary license ends when the medicinal product is actually supplied^31^	Not applicable^31^	No timeline, as soon as possible^31^	National (inspectorate on cantonal level)^32^	Preliminary opinion from the ethics committee^31^ Justification treatment centers^31^ Medicine is identical to the medicine used in the clinical trial approved by Swissmedic and the ethics committee^31^	Yes^31^
UK	The early access to medicines scheme^33^	MHRA^33^	Manufacturer^34^	1 Year^35^	Yes^33^	45 Days^33^	National^33^	Promising innovative medicine designation^33^	Yes^33^

ANSM, Agence Nationale de Sécurité du Médicament et des Produits de Santé; BfArm, Bundesinstitut für Arzneimittel und Medizinprodukte; CBG, College ter Beoordeling van Geneesmiddelen; FAGG, Federaal Agentschap voor Geneesmiddelen en Gezondheidsproducten; HAS, Haute Autorité de Santé; IGJ, Inspectie Gezondheidzorg en Jeugd; MHRA, Medicines and Healthcare products Regulatory Agency; NOMA, Norwegian Medical Products Agency; PEI, Paul-Ehrlich-Institut (Agency of the *German* Federal Ministry of Health).

Timelines for CUPs varied across the countries, with assessment periods ranging from 14 to 60 days.^22^^,^^23^^,^^25^^,^^27^, ^28^, ^29^^,^^33^ In all countries studied, except Belgium, CUPs were valid for a duration of 1 year,^23^^,^^25^^,^^27^, ^28^, ^29^^,^^35^ with extensions permitted in all countries but Germany.^23^^,^^27^^,^^29^ In Belgium, however, CUPs had no predetermined duration and remained valid until the medicine became commercially available and reimbursed^21^ [A Schiel (personal communication), A Zenner (personal communication)]. In Switzerland, the ‘temporary authorization’ automatically converted into a regular MA and reimbursement upon completion of the final MA assessment and when the medicine was actually supplied.^31^

### Regulatory characteristics of NPBs

In four countries, Belgium, France, the Netherlands, and Norway, NPBs were valid for 1 year. In six countries, NPBs granted access for individual patients^46^, ^38^, ^40^, ^41^, ^48^, ^42^, ^54^, ^47^ [A Zenner (personal communication), A Berger (personal communication)] (Table 3). In Norway, however, NPBs could also be granted to individual clinics or hospitals^48^; in the Netherlands, they could be issued per indication^46^; and in Belgium, they could be assigned per patient group to a single prescriber^38^ [A Zenner (personal communication)].

**Table 3:** Overview of routing for named patient basis in seven European countries

Country	Name program	Responsible authority	Who applies	Duration	Extension possible	Assessment period	On what level	Additional requirements	Fee to the authority
Belgium	Artsenverklaring^36^	Pharmacist and physician^36^^,^^37^ [A Zenner (personal communication)]	Physician^38^	1 Year^38^	Not specified [A Zenner (personal communication)]	Not specified [A Zenner (personal communication)]	Individual patient (public pharmacy) or a group of patients (hospital pharmacy)^38^ [A Zenner (personal communication)]	The pharmacist needs a prescription and a doctor’s declaration [A Zenner (personal communication)]	N/A [A Zenner (personal communication)]
France	Access compassionnel^39^	ANSM^40^	Physicians, pharmacists^40^	1 Year or until max. 3 months after authorization^40^^,^^41^	Not specified	Not specified	Individual patient^40^	N/A	N/A
Germany	Individuelle Heilversuche^42^	Ethics committee^43^^,^^44^	Physician must request at company^42^	Unregulated	Unregulated	Unregulated	Individual patient^42^ (<10, not legally defined) [A Berger (personal communication)]	Patient consent,^42^^,^^45^ physician must have experience with diagnosis (ref) and thoroughly document the case [A Berger (personal communication)]	N/A
Netherlands	Leveren op artsenverklaring^46^	IGJ^46^	Manufacturer, wholesale distributor, (hospital) pharmacist, general practitioner with pharmacy^46^	1 Year^46^	Yes^46^	Max. 8 weeks^46^	Individual patient (indication level possible)^46^	N/A	N/A
Norway	Named patient^47^	NOMA^47^	Doctor or dentist^47^	Max. 1 year^48^	Need to file a new application^49^	Usually three working days^48^	Individual patient, practice or hospital^48^	Hospital management must approve^50^	N/A
Switzerland	Heilversuch^51^	Cantonal authorities^52^	Private individuals for personal use or medical professionals^47^	Unregulated	Unregulated	Unregulated	Small quantities^47^	Patient consent^51^	N/A
UK	Named patient or ‘specials’^53^	MHRA^53^	Prescriber,^54^^,^^53^^,^^55^ doctor, dentist, nurse independent prescriber, pharmacist independent prescriber, supplementary prescriber^54^^,^^55^	Unspecified [D O'Connor (personal communication)]	Unspecified [D O'Connor (personal communication)]	Unspecified [D O'Connor (personal communication)]	Individual patient^54^	Approval from clinical director^53^	N/A

ANSM, Agence nationale de sécurité du médicament et des produits de santé; HAS, Haute Autorité de santé; IGJ, Inspectie Gezondheidszorg en Jeugd; max, maximum; MHRA, Medicines and Healthcare products Regulatory Agency; NOMA, Norwegian Medical Products Agency.

The organizations authorized to grant NPBs could be either centralized or decentralized. In France, Norway, and the UK, NCAs responsible for assessing MA applications handled the NPB applications.^40^^,^^50^^,^^53^ In the Netherlands, the national inspectorate handled the procedure.^46^ In Germany, decentralized responsibility lay with local ethics committees, while in Switzerland, it fell to cantonal inspectorates.^43^^,^^44^^,^^52^ Since Belgium had no national procedures for NPBs, individual pharmacies handle them^36^^,^^37^ [A Zenner (personal communication)]. Typically, applicants for NPBs are health care professionals such as physicians or pharmacists, with the Netherlands being the only country where manufacturers can directly apply for NPBs.^46^, ^38^, ^40^^,^^42^, ^54^, ^47^^,^^50^^,^^53^^,^^55^

### Reimbursement for CUPs

Further investigations focused on the regulations concerning charging costs for medicinal products in CUPs (Table 4). In Belgium, Germany, and Norway, charging patients was prohibited by law.^21^^,^^26^^,^^29^ In Norway, however, case-by-case payment agreements could be negotiated between the manufacturer, the responsible institution, and the pharmacy.^29^ In France and Switzerland, charging patients for medicines in CUPs was not prohibited^34^ [Projektgruppe Einzelfallvergütung (personal communication)]. In the Netherlands and the UK, charging patients for medicines in CUPs is considered unacceptable.^56^

**Table 4:** Overview of reimbursement possibilities for compassionate use programs in seven European countries

Country	National reimbursement possibilities	Reimbursement organization	Reimbursement applicant	Payer	Charging patient allowed	Other costs (such as outpatient stay or hospitalization)
Belgium	Yes^57^	RIZIV-INAMI^21^	Manufacturer or minister of Social Affairs^60^	If decision: RIZIV-INAMI^60^	No (prohibited)^21^	Patient^57^
France	Yes^58^^,^^59^	Health insurance^58^^,^^59^	Not applicable (automatically reimbursed)^58^^,^^59^	Health insurance^58^^,^^59^	Unspecified	Unspecified
Germany	No^26^	Not applicable	Not applicable	Manufacturer	No (prohibited)^26^	Health insurance (other treatment costs and possible hospitalization)^61^
Netherlands	No^56^	Not applicable^56^	Not applicable^56^	Manufacturer^62^	No (not prohibited)^56^	Health insurance
Norway	No^29^	Not applicable	Not applicable	Manufacturer, the responsible institution and/or the pharmacy^29^	No (prohibited)^29^	Unspecified
Switzerland	Unregulated	Unregulated [Projektgruppe Einzelfallvergütung (personal communication)]	Unregulated. [Projektgruppe Einzelfallvergütung (personal communication)]	Unregulated [Projektgruppe Einzelfallvergütung (personal communication)]	Unregulated [Projektgruppe Einzelfallvergütung (personal communication)]	Unregulated [Projektgruppe Einzelfallvergütung (personal communication)]
UK	No^34^	Not applicable	Not applicable	Manufacturer^34^	No^34^	Medicines are provided free of charge by the manufacturer^34^ Other costs may be borne by the health system (e.g. diagnostics) [D O'Connor (personal communication)]

RIZIV, Rijksinstituut voor ziekte- en invaliditeitsverzekering.

Reimbursement options for the medicines in CUPs were absent in most countries, except for Belgium and France.^26^^,^^29^^,^^34^^,^^57^^,^^58^^,^^59^^,^^56^ In Belgium, manufacturers or the Minister of Social Affairs could apply for a ‘cohort application’ at the national reimbursement organization (Rijksinstituut voor Ziekte- en invaliditeitsverzekering, RIZIV), which was evaluated by a Board of Physician Directors.^21^^,^^60^ Afterwards, an individual request must be submitted for each patient. In France, reimbursement is contingent upon several criteria: (i) absence of appropriate treatments for the specific indication; (ii) necessity of starting treatment without delay; (iii) strong assumption of safety and efficacy based on clinical trial results; and (iv) pre-authorization medicine being considered innovative, particularly in comparison to clinically relevant alternatives.^58^^,^^59^

Apart from reimbursement options for medicines in CUPs, coverage for additional costs related to side-effect management, hospitalization, outpatient care and/or hospital pharmacy expenses, was generally not regulated. In Germany, however, health care insurances covered these expenses. In the Netherlands, only hospitalization costs and additional treatments due to adverse events were covered, while in Belgium, patients were responsible for bearing these costs^57^^,^^61^^,^^63^ [D O’Connor (personal communication)].

### Reimbursement for NPBs

For medicines provided through NPBs, limited information was available on charging costs, reimbursement, and coverage of additional costs. It appears that charging patients for medicines provided under NPBs is not explicitly forbidden (Table 5).^42^^,^^53^^,^^64^^,^^65^ In Germany and Switzerland, the charging and reimbursement of NPBs were unregulated^42^ [A Berger (personal communication), Projektgruppe Einzelfallvergütung (personal communication)]. In other countries, reimbursement for NPBs may only be granted on an exceptional basis.^48^^,^^66^^,^^67^, ^68^, ^69^^,^^70^^,^^71^^,^^72^ Since these exceptions for reimbursement usually required safety and efficacy to be established, they tended to focus on other NPB applications, such as the import of medicines authorized in third countries.

**Table 5:** Overview of reimbursement possibilities for named patient basis in seven European countries

Country	National reimbursement possibilities	Reimbursement organization	Who applies for reimbursement?	Who pays	Charging patient for NPB	Other costs (such as outpatient stay or hospitalization)
Belgium	On exceptional basis for rare diseases without alternatives^66^	RIZIV^64^	Patient^73^	Patient, health insurance or Bijzonder solidariteitsfonds^66^^,^^64^	Allowed^64^	Unspecified
France	Yes^67^, ^68^, ^69^	HAS^74^	Health care facility^74^	HAS^74^	See CUP^74^	HAS^74^
Germany	Unregulated^42^ [A Berger (personal communication)]	N/A	N/A	Covered in the in-patient setting or assessment on case-by-case basis. All parties involved: patient, physician, company, and health insurance^42^ [A Berger (personal communication)].	Allowed^42^	Unregulated
Netherlands	On exceptional basis: in case of rational pharmacology and a prevalence of <1 : 150 000^70^^,^^71^	Health insurances^65^	Physician^75^	Manufacturer^71^	No (not prohibited)^65^	Health insurance or hospital
Norway	Individual benefits for expenses for exempted products can be granted^48^	HELFO^48^	Prescribing doctor^48^	If granted: HELFO^48^	Unspecified^50^	If granted: HELFO^48^
Switzerland	On request^72^	Health insurance^72^	Unregulated [Projektgruppe Einzelfallvergütung (personal communication)]	If request is granted: health insurance^72^	Unregulated [Projektgruppe Einzelfallvergütung (personal communication)]	Unregulated [Projektgruppe Einzelfallvergütung (personal communication)]
UK	Unspecified [D O'Connor (personal communication)]	Unspecified [D O'Connor (personal communication)]	Unspecified [D O'Connor (personal communication)]	Manufacturer or patient^53^	Not prohibited^53^	Unspecified [D O'Connor (personal communication)]

CUP, compassionate use program; HAS, Haute Autorité de santé; HELFO, The Norwegian Health Economics Administration; NPB, named patient basis; RIZIV, Rijksinstituut voor ziekte- en invaliditeitsverzekering.

### In-depth examination of CUPs for anticancer and orphan medicines authorized in 2021 and 2022

We conducted an in-depth examination of the CUPs of the anticancer and orphan medicines EU authorized in 2021 and 2022. A flow diagram outlining the selection procedure is provided in Supplementary Figure S2, available at https://doi.org/10.1016/j.esmoop.2025.105855. Between January 2021 and December 2022, 20 anticancer non-orphan medicines, 12 anticancer orphan medicines, and 27 non-anticancer orphan medicines received EU-MA (Supplementary Table S2, available at https://doi.org/10.1016/j.esmoop.2025.105855). In the UK, only four medicines (asciminib, sacituzumab govitecan, sotorasib, and tepotinib) received UK-MA before EU-MA with a maximum difference of 4 months (Supplementary Table S1, available at https://doi.org/10.1016/j.esmoop.2025.105855). In total, 59 medicines were evaluated for availability through CUPs in the selected countries.

**Figure 1:**
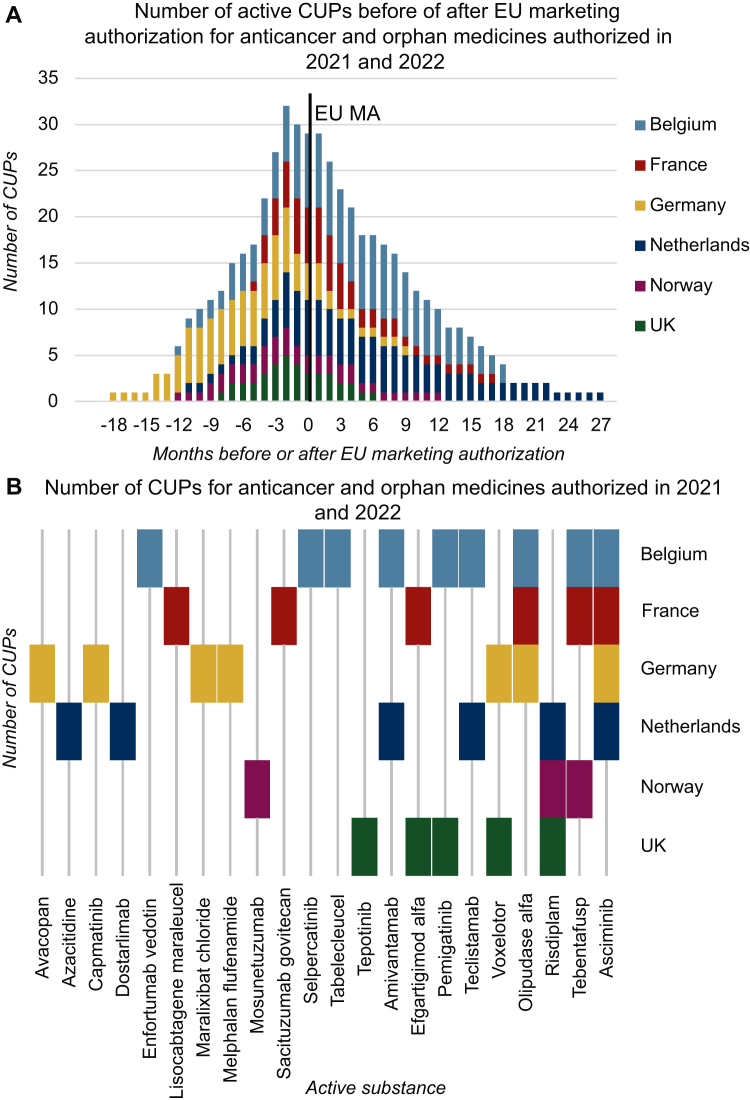
**Number of compassionate use programs (CUPs).** (A) Number of active CUPs before and after European Union (EU) marketing authorization (EU-MA) for anticancer and orphan medicines authorized in 2021 and 2022. Out of a total sample of 59 medicines, 22 were made available through 36 CUPs across the countries studied, with variable durations. The *x*-axis shows the number of months before and after the EU-MA (month = 0, highlighted by the black line). The *y*-axis displays the number of active CUPs. The figure illustrates that many CUPs start before EU-MA and last several months after MA. UK-MA was granted after EU-MA for all medicines with a CUP, except for asciminib, sacituzumab govitecan, and tepotinib. (B) Number of CUPs available for anticancer and orphan medicines authorized in 2021 and 2022. Out of a total sample of 59 medicines, 22 were made available through 36 CUPs across the countries studied. The *x*-axis shows the number of CUPs available per country, either before or after MA. The *y*-axis displays the number of CUPs available per medicine. The figure highlights that Belgium, France, Germany, and the Netherlands had more CUPs than Norway and the UK. Furthermore, the figure illustrates that 13 medicines had one CUP each, 5 medicines had two CUPs, 3 medicines had three CUPs, and 1 medicine was available through four CUPS.

For 22 medicines of the 59 medicines in the sample, CUPs were identified (37%, Table 6). These included 11 of the 20 anticancer non-orphan medicines (55%), 5 of the 12 anticancer orphan medicines (42%), and 6 of the 27 non-anticancer orphan medicines (22%). In total, 36 CUPs were identified across the seven countries in the sample (Figure 1). Of the 17 conditional MAs, 6 were made available through a CUP (35%). Of the 11 MAs under exceptional circumstances, 3 were accessible through a CUP (27%). Additionally, of the 13 medicines with PRIME status, 7 were available through a CUP (54%).

**Table 6:** Start and end dates of compassionate use programs (CUPs)

Tradename	Active substance	Anticancer (A) /orphan(O) medicine	Belgium	France	Netherlands	Germany	Norway	UK
Abecma	Idecabtagene vicleucel	AO	N/A	N/A	N/A	N/A	N/A	N/A
Amvuttra	Vutrisiran	O	N/A	N/A	N/A	N/A	N/A	N/A
Artesunate Amivas	Artesunate	O	N/A	N/A	N/A	N/A	N/A	N/A
Aspaveli	Pegcetacoplan	O	N/A	N/A	N/A	N/A	N/A	N/A
Breyanzi	Lisocabtagene maraleucel	A	N/A	8 September 2022 until 20 September 2023	N/A	N/A	N/A	N/A
Brukinsa	Zanubrutinib	A	N/A	N/A	N/A	N/A	N/A	N/A
Bylvay	Odevixibat	O	N/A	N/A	N/A	N/A	N/A	N/A
Carvykti	Ciltacabtagene autoleucel	AO	N/A	N/A	N/A	N/A	N/A	N/A
Copiktra	Duvelisib	A	N/A	N/A	N/A	N/A	N/A	N/A
Ebvallo	Tabelecleucel	AO	17 March 2023 until 17 April 2024	N/A	N/A	N/A	N/A	N/A
Elzonris	Tagraxofusp	AO	N/A	N/A	N/A	N/A	N/A	N/A
Enhertu	Trastuzumab Deruxtecan	A	N/A	N/A	N/A	N/A	N/A	N/A
Enjaymo	Sutimlimab	O	N/A	N/A	N/A	N/A	N/A	N/A
Enspryng	Satralizumab	O	N/A	N/A	N/A	N/A	N/A	N/A
Evrysdi	Risdiplam	O	N/A	N/A	23 October 2020 until 1 July 2023	N/A	8 May 2020 until 19 February 2021	17 September 2020 until 1 July 2021
Filsuvez	Birch bark extract	O	N/A	N/A	N/A	N/A	N/A	N/A
Gavreto	Pralsetinib	A	N/A	N/A	N/A	N/A	N/A	N/A
Imcivree	Setmelanotide	O	N/A	N/A	N/A	N/A	N/A	N/A
Inrebic	Fedratinib	O	N/A	N/A	N/A	N/A	N/A	N/A
Jemperli	Dostarlimab	A	N/A	N/A	4 March 2021 until 3 March 2023	N/A	N/A	N/A
Kimmtrak	Tebentafusp	AO	21 September 2021 until 1 November 2023	27 January 2022 until 4 January 2023	N/A	N/A	16 January 2022 until 1 May 2023	N/A
Koselugo	Selumetinib	O	N/A	N/A	N/A	N/A	N/A	N/A
Livmarli	Maralixibat chloride	O	N/A	N/A	N/Aa	24 February 2022 until 24 February 2023	N/A	N/A
Livtencity	Maribavir	O	N/A	N/A	N/A	N/A	N/A	N/A
Lumoxiti	Moxetumomab pasudotox	AO	N/A	N/A	N/A	N/A	N/A	N/A
Lumykras	Sotorasib	A	N/A	N/A	N/A	N/A	N/A	N/A
Lunsumio	Mosunetuzumab	AO	N/A	N/A	N/A	N/A	14 October 2021 until 21 October 2022	N/A
Minjuvi	Tafasitamab	AO	N/A	N/A	N/A	N/A	N/A	N/A
Nexpovio	Selinexor	A	N/A	N/A	N/A	N/A	N/A	N/A
Ngenla	Somatrogon	O	N/A	N/A	N/A	N/A	N/A	N/A
Nulibry	Fosdenopterin	O	N/A	N/A	N/A	N/A	N/A	N/A
Onureg	Azacitidine	A	N/A	N/A	12 March 2021 until 11 March 2022	N/A	N/A	N/A
Opdualag	Relatlimab/nivolumab	A	N/A	N/A	N/A	N/A	N/A	N/A
Oxbryta	Voxelotor	O	N/A	N/A	N/A	26 April 2021 until 26 April 2022	N/A	25 January 2022 until 25 July 2022
Padcev	Enfortumab vedotin	A	22 August 2022 until 22 March 2023	N/A	N/A	N/A	N/A	N/A
Pemazyre	Pemigatinib	AO	22 January 2021 until 1 July 2022	N/A	N/A	N/A	N/A	15 January 2021 until 7 April 2021
Pepaxti	Melphalan flufenamide	A	N/A	N/A	N/A	12 March 2021 until 12-March 2022	N/A	N/A
Pyrukynd	Mitapivat	O	N/A	N/A	N/A	N/A	N/A	N/A
Qinlock	Ripretinib	AO	N/A	N/A	N/A	N/A	N/A	N/A
Retsevmo	Selpercatinib	A	4 January 2021 until 1 March 2022	N/A	N/A	N/A	N/A	N/A
Roctavian	Valoctocogene roxaparvovec	O	N/A	N/A	N/A	N/A	N/A	N/A
Rybrevant	Amivantamab	A	11 June 2021 until 11 July 2023	N/A	29 October 2021 until 1 April 2023	N/A	N/A	N/A
Scemblix	Asciminib	AO	27 September 2021 until 1 May 2023	14 April 2022 until 23 November 2022	2 November 2021 until 2 November 2022	11 October 2021 until 11 October 2022	N/A	N/A
Skysona	Elivaldogene autotemcel	O	N/A	N/A	N/A	N/A	N/A	N/A
Skytrofa (previously Lonapegsomatropin Ascendis Pharma)	Lonapegsomatropin	O	N/A	N/A	N/A	N/A	N/A	N/A
Sogroya	Somapacitan	O	N/A	N/A	N/A	N/A	N/A	N/A
Tabrecta	Capmatinib	A	N/A	N/A	N/A	31 May 2021 until 31 May 2022	N/A	N/A
Tavneos	Avacopan	O	N/A	N/A	N/A	16 November 2021 until 16 November 2022	N/A	N/A
Tecvayli	Teclistamab	A	12 August 2022 until 1 September 2023	N/A	22 July 2022 until 22 July 2023	N/A	N/A	N/A
Tepmetko	Tepotinib	A	N/A	N/A	N/A	N/A	N/A	12 July 2021 until 24 September 2021
Trodelvy	Sacituzumab govitecan	A	N/A	6 September 2021 until 6 April 2022	N/A	N/A	N/A	N/A
Tukysa	Tucatinib	A	N/A	N/A	N/A	N/A	N/A	N/A
Upstaza	Eladocagene exuparvovec	O	N/A	N/A	N/A	N/A	N/A	N/A
Voraxaze	Glucarpidase	O	N/A	N/A	N/A	N/A	N/A	N/A
Voxzogo	Vosoritide	O	N/A	N/A	N/A	N/A	N/A	N/A
Vyvgart	Efgartigimod alfa	O	N/A	21 July 2022 until 14 December 2022	N/A	N/A	N/A	25 May 2022 until 14 March 2023
Xenpozyme	Olipudase alfa	O	27 September 2021 until 27 April 2023	22 March 2022 until 23 November 2022	N/A	1 June 2021 until 1 June 2022	N/A	N/A
Zokinvy	Lonafarnib	O	N/A	N/A	N/A	N/A	N/A	N/A
Zynlonta	Loncastuximab tesirine	A	N/A	N/A	N/A	N/A	N/A	N/A

N/A, no CUPs available.

Most CUPs commenced before EU-MA (*n* = 32, 83%) (Table 7, Figure 1A). On average, CUPs began 145 days before EU-MA, with the earliest starting 523 days before EU-MA, and the latest starting 157 days after EU-MA (median: 96 days before EU-MA). The end dates of the CUPs varied, with some concluding before EU-MA and others continuing afterward. On average, CUPs lasted 246 days after EU-MA, with the first ending 158 days before EU-MA, and the longest lasting 827 days after EU-MA (median: 233 days after EU-MA). The overall average duration of the CUPs was 391 days, with a range from a minimum of 74 days to a maximum of 981 days (median: 365 days).

**Table 7:** Duration of compassionate use programs

Country	Trade name	Active substance	Anticancer (A)/orphan medicine (O)	Days before (−) or after EU-MA at CUP start	Days before (-) or after EU-MA at CUP end	Total CUP duration (days)
Belgium	Ebvallo	Tabelecleucel	AO	91	457	366
	Kimmtrak	Tebentafusp	AO	−192	579	771
	Padcev	Enfortumab vedotin	A	131	343	212
	Pemazyre	Pemigatinib	AO	−63	462	525
	Retsevmo	Selpercatinib	A	−38	383	421
	Rybrevant	Amivantamab	A	−181	579	760
	Scemblix	Asciminib	AO	−332	249	581
	Tecvayli	Teclistamab	A	−11	374	385
	Xenpozyme	Olipudase alfa	O	−270	307	577
	*Mean*			*−96*	*415*	*511*
	*Median*			*−63*	*383*	*525*
France	Breyanzi	Lisocabtagene Maraleucel	A	157	534	377
	Kimmtrak	Tebentafusp	AO	−64	278	342
	Scemblix	Asciminib	AO	−133	90	223
	Trodelvy	Sacituzumab Govitecan	A	−77	135	212
	Vyvgart	Efgartigimod alfa	O	−20	126	146
	Xenpozyme	Olipudase alfa	O	−94	152	246
	*Mean*			*−39*	*219*	*258*
	*Median*			*−71*	*143*	*235*
Germany	Livmarli	Maralixibat chloride	O	−288	77	365
	Oxbryta	Voxelotor	O	−294	71	365
	Pepaxti	Melphalan Flufenamide	A	−523	−158	365
	Scemblix	Melphalan Flufenamide	AO	−318	47	365
	Tabrecta	Capmatinib	A	−385	−20	365
	Tavneos	Avacopan	O	−56	309	365
	Xenpozyme	Olipudase alfa	O	−388	−23	365
	*Mean*			*−322*	*43*	*365*
	*Median*			*−318*	*47*	*365*
Netherlands	Evrysdi	Risdiplam	O	−154	827	981
	Jemperli	Dostarlimab	A	−48	681	729
	Onureg	Azacitidine	A	−97	267	364
	Rybrevant	Amivantamab	A	−41	478	519
	Scemblix	Asciminib	AO	−296	69	365
	Tecvayli	Teclistamab	A	−32	333	365
	*Mean*			*−111*	*442*	*554*
	*Median*			*−73*	*405*	*442*
Norway	Evrysdi	Risdiplam	O	−322	−35	287
	Kimmtrak	Tebentafusp	AO	−75	395	470
	Lunsumio	Mosunetuzumab	AO	−232	140	372
	*Mean*			*−210*	*167*	*376*
	*Median*			*−232*	*140*	*372*
United Kingdom	Evrysdi	Risdiplam	O	−190	97	287
	Oxbryta	Voxelotor	O	−20	161	181
	Pemazyre	Pemigatinib	AO	−70	12	82
	Tepmetko	Tepotinib	A	−219	−145	74
	Vyvgart	Efgartigimod alfa	O	−77	216	293
	*Mean*			*−115*	*68*	*183*
	*Median*			*−77*	*97*	*181*
Overall mean				−145	246	391
Overall median				−96	233	365

A, anticancer; CUP, compassionate use program; MA, marketing authorization; O, orphan medicine.

**Figure 2:**
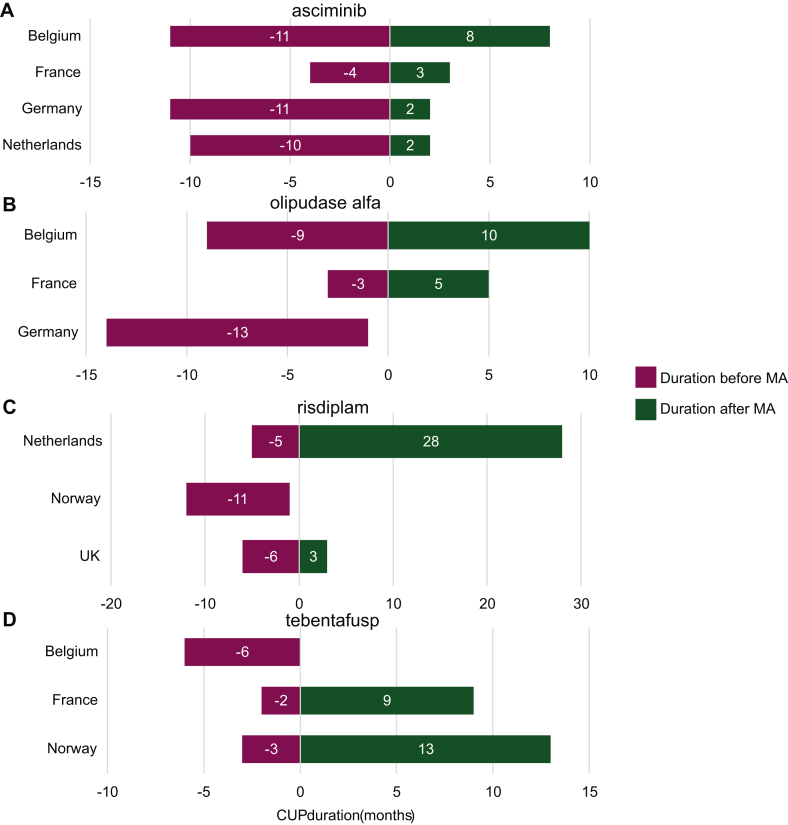
**Duration of compassionate use programs (CUPs) available in at least three countries.** Purple represents the number of months before European Union (EU) marketing authorization (MA), while green represents the number of months after EU-MA. (A) Duration of CUPs for asciminib (Scemblix) in Belgium, France, Germany, and the Netherlands. In France, the CUP started later, while in Belgium, the CUP lasted longer. (B) Duration of CUPs for olipudase alfa (Xenpozyme) in Belgium, France, and Germany. In France, the CUP started later than in the other countries. In Germany, the CUP ended before MA. (C) Duration of CUPs for risdiplam (Evrysdi) in the Netherlands, Norway, and the UK. In Norway, the CUP started earlier than in the other countries and ended before MA. In the Netherlands, the CUP lasted longer: until 28 months after MA. (D) Duration of CUPs for tebentafusp (Kimmtrak) in Belgium, France, and Norway. In Belgium, the CUP started earlier than in the other countries and ended at MA. In Norway and France, the CUP continued after MA, until 9 and 13 months, respectively.

Among the selected countries, the availability of CUPs varied. Belgium had the highest number, with nine CUPs, seven of which focused on cancer. Two of these CUPs commenced after EU-MA. In Belgium, the CUPs can continue until medicines are reimbursed. France had six CUPs, with durations ranging from 145 to 377 days. Germany reported seven CUPs, mostly for non-anticancer orphan medicines (*n* = 4), including two that started >1 year before EU-MA and lasted 365 days. The Netherlands had six CUPs, primarily for anticancer medicines (*n* = 5), with durations ranging from 365 to 981 days. Norway had three CUPs, lasting between 287 and 470 days. The UK had five CUPs, with durations ranging from 74 to 293 days. If CUPs were available after EU-MA, they ended at UK-MA. One CUP received UK-MA before EU-MA (tepotinib).

Most CUPs were available in one or two countries (Figure 1B). CUPs for risdiplam for 5q spinal muscular atrophy and tebentafusp for uveal melanoma were available in three countries, while asciminib for chronic myeloid leukemia was available in four countries, with durations varying from 246 to 981 days (Figure 2).

Belgium and France were the only countries that offered reimbursement options for CUPs. In Belgium, none of the CUPs were included on the RIZIV’s reimbursement list.^60^ In France, reimbursement of the CUPs was incorporated into the application process.

## Discussion

We found variable regulatory characteristics across the countries in the sample, which are generally more stringent for CUPs and often less clearly defined for NPBs. For example, national regulations generally outlined whether charging costs for CUPs are permitted and, if so, who is responsible for covering them, often stipulating that patients should not bear these expenses. In contrast, regulations governing cost charging and reimbursement aspects for NPBs were less clearly defined, highlighting a distinction between the two routes. Our findings reveal that most countries lacked formal NPB reimbursement mechanisms. If reimbursement was possible, it was generally restricted to exceptional conditions for *ad hoc* import of medicines with established safety and efficacy and may thus not apply to pre-authorization access. This regulatory ambiguity can impose administrative burdens on physicians and create financial barriers for patients, particularly those with limited resources, contributing to inequality in access.

We carried out an in-depth examination of EU-authorized medicines made available through CUPs, which indicates differences between countries in both the number and duration of these programs. Of the 59 authorized medicines, only 22 were granted CUPs. This was particularly limited for non-anticancer orphan medicines (22%), compared with anticancer non-orphan medicines (55%), reflecting that CUP eligibility is dependent on the clinical indication. When CUPs were available, access was often confined to a limited number of countries, generally one or two, indicating unequal access to medicines through CUPs across Europe. Notably, only one medicine, asciminib, was available in four countries, with the duration varying considerably from 246 to 981 days. Beyond these cross-country differences, we observed instances where CUPs extended beyond MA, with the longest extending 827 days after authorization. This raises questions about the consistency and intended purpose of CUPs. It is important to note, however, that both the reported availability and its duration may be underestimated, as some medicines could be provided through NPBs, which are not publicly disclosed.

This study focused more extensively on CUPs due to data availability, yet NPBs represent another important route for accessing medicines. Per country, there may be local differences in how these pathways are used. The lack of (publicly) available and centralized data, however, limits data collections and analysis.

These (cross-country) disparities could make patients feel compelled to seek treatment abroad, potentially exposing them to medicines that have not undergone comprehensive benefit–risk assessment. This raises safety concerns, especially when health care providers in the patient’s home country lack familiarity with the unauthorized medicines. Besides, patients bearing the costs themselves raises ethical concerns, given that only those with greater financial resources can afford such care.^76^ Several other concerns were raised by Bunnik and Aarts.^77^ For instance, physicians often experience resistance toward pre-authorization access, driven by their desire to avoid giving false hope to patients.^77^ Practical hurdles, such as the time, costs, and effort required for these processes, the chance of reimbursement refusal as well as concerns about charging patients, may contribute to their hesitance.^77^ Harmonizing access through CUPs and NPBs across Europe mitigates these risks and reduces the reliance on intermediaries such as myTomorrows and everyone.org.^78^^,^^79^

Although two countries, Belgium and France, offered reimbursement options for medicines accessed through CUPs, the number of CUPs was comparable to Germany and the Netherlands, where no reimbursement options existed. This suggests that reimbursement options do not directly increase the number of CUPs. While variability was observed among the five EU countries, the generalizability to broader pre-authorization access across Europe remains limited. Nonetheless, variations in CUP availability are likely to exist across Europe.

While we recognize that including lower-income countries would provide a more comprehensive understanding of CUP and NPB challenges, in this study we focused on countries with relatively similar economic levels and comparable health care systems. For example, in Spain, CUPs exist, but a decentralized health care system with regionally managed budgets hinders analysis and cross-country comparison.^80^^,^^81^ In Poland, attempts have been made to implement CUPs, but formal regulation is still absent.^82^ In Italy, several programs exist with notable regional differences, and pharmaceutical companies are typically expected to cover the costs of treatment for medicines without a negotiated price (which can mean both before and after EU-MA).^80^^,^^83^ Nonetheless, considerable variations in CUPs are likely to exist across—and potentially even within—all 27 countries.

Our study offers several novel contributions. To our knowledge, this is the first multinational policy analysis to examine CUPs in NPBs concurrently, incorporating charging and reimbursement aspects. Unlike previous studies, we systematically assessed access through CUPs for 59 recently authorized medicines, providing a more comprehensive understanding of the variation across Europe. For instance, prior studies mainly focused on CUPs and relied on surveys and multidisciplinary panel workshops, which point out the need for a more formal assessment of the data and decision support in the approval process of a CUP application.^84^^,^^85^

Our findings align with the conclusions of Tarantola et al.,^80^ who noted that the variety of pre-authorization access routes may not promote equitable access. Information on NPBs is often undisclosed, which may lead to some physicians lacking awareness on the full range of available medicines.^86^ This can result in some patients gaining access to a medicine through their physicians, while others do not.^86^ Publicly available lists of medicines provided by NPBs—similarly to CUPs—could better inform physicians, patients, and the general public.^87^ This would enable an in-depth examination of access to medicines through NPBs across Europe. To prevent unrealistic expectations, however, it is important to ensure that unauthorized medicines are not overly advertised.^86^ Hence, we believe that manufacturers should focus on CUPs, rather than NPBs. Importantly, rather than advocating more CUPs, we argue that the focus should be on improving transparency and reducing variability. The upcoming revision of European pharmaceutical legislation provides an opportunity to address these challenges.^88^

When comparing our findings with studies on reimbursement timelines, it is not surprising that CUPs tend to have longer durations in countries with lengthy reimbursement processes.^7^ As a result, manufacturers may become reluctant to provide medicines free-of-charge before MA, as recently demonstrated by manufacturers Servier and Merck Sharp & Dohme, which announced that they would not do this.^89^^,^^90^ Interestingly, in countries where charging costs are allowed, prices during the pre-authorization phase were found to be higher compared with post-authorization prices.^15^

In this context, new managed access programs starting before MA could help prevent avoiding inequality in access to pre-authorized medicines. These programs should involve multiple stakeholders, including physicians, manufacturers, payers, patient organizations, and regulatory authorities.^15^^,^^91^ Ideally, these programs facilitate real-world data collection for MA application and HTA, retroactive reimbursement, and adaptive medicine pricing strategies.^86^^,^^92^^,^^93^ Platforms with national coordination centers like the Dutch (Orphan) Drug Access Protocol (O)DAP, could help facilitate timely and controlled access.^94^^,^^95^

This study has several limitations. Firstly, the analysis relies heavily on publicly available data and expert consultations, which may not fully capture the complete landscape, particularly for NPBs. Secondly, the inclusion of only seven high-income European countries limits the generalizability of the findings to the broader EU context. Lastly, the findings represent a cross-sectional snapshot, and may not account for recent or ongoing regulatory reforms. Furthermore, it is worth noting that regulations may have changed since the data were collected. For instance, in the Netherlands, a change in the CUP duration was announced in December 2024, with CUPs now granted until the medicine receives MA, rather than the previous 1 year duration. Additionally, gene therapies are now eligible.^96^

Several avenues for future research can be identified. First, it is important to explore new approaches to stimulate timely access, in which uncertainties are reduced, thereby enabling data collection for a robust assessment of safety and efficacy and facilitate reasonable pricing and reimbursement aligning with the remaining uncertainties over time. Besides, future studies should investigate the impact of varying national policies throughout the EU, access patterns on patient decision-making, as well as the potential use of data from CUPs and NPBs in pricing and reimbursement negotiations. Moreover, scrutinizing patient outcomes and experiences is essential to better understand their safety, effectiveness, and impact of CUPs and NPBs. Additionally, further research is needed to better understand the motivations and barriers faced by stakeholders in providing medicines through CUPs or NPBs.

Overall, CUPs and NPBs provide a last resort option for patients with chronically debilitating or life-threatening diseases who lack authorized therapies or cannot participate in clinical trials.^11^ Careful monitoring is needed, however, to prevent unnecessary exposure to unauthorized medicines with uncertain benefit–risk profiles.

### Conclusions

This study provided an overview of the regulatory characteristics of CUPs and NPBs, including charging of costs and reimbursement aspects, in seven European countries. We observed substantial variability in the implementation of the EU regulation governing CUPs and NPBs, with CUPs generally being more tightly regulated than NPBs. Our in-depth examination of CUPs for orphan and anticancer medicines authorized in 2021 and 2022 demonstrated differences in both availability and duration across countries. The lack of transparency regarding which NPBs are available before EU-MA limits awareness on available medicines and may therefore compromise equitable access. To address these challenges, and improve access to promising and innovative medicines for patients with unmet medical needs, minimizing disparities across pre-authorization access routes across Europe is recommended.
