# Supplementary material for: Access to anticancer and orphan medicines through compassionate use programs and named patient basis in seven European countries
Source: ESMO Open. 2025 Nov 13;10(11):105855. doi: 10.1016/j.esmoop.2025.105855 (PMC12661435; doi:10.1016/j.esmoop.2025.105855)

**Supplements** Access to anticancer and orphan medicines through compassionate use programs and named patient basis in seven European countries

**Supplementary Table S1.** Total sample of anticancer and orphan medicines granted EU marketing authorization in 2021 and 2022. All data were European Public Assessment Report (EPAR) databases (downloaded July 2023), supplemented with data from the European Medicines Regulatory Database (EMRD) (downloaded February 2025), and with data on the marketing authorization date in United Kingdom and CUP availability in Belgium, France, Germany, the Netherlands, Norway, or the United Kingdom. EU: European Union. MA: Marketing authorization. UK: United Kingdom. MAH: Marketing authorization holder. O: Orphan medicine at the time of the granting of the marketing authorization. AA: Accelerated assessment. CA: Conditional marketing authorization. EC: Marking authorization under exceptional circumstances. PRIME: Priority medicine. CUP: Compassionate use program.

| **Trade name** | **Active substance** | **Initial indication** | **EU MA date** | **UK MA date** | **Initial MAH** | **O** | **AA** | **CA** | **EC** | **PRIME** | **CUP** |
| --- | --- | --- | --- | --- | --- | --- | --- | --- | --- | --- | --- |
| Abecma | idecabtagene vicleucel | Treatment of adult patients with relapsed and refractory multiple myeloma who have received at least three prior therapies, including an immunomodulatory agent, a proteasome inhibitor and an anti CD38 antibody, and have demonstrated disease progression on the last therapy. | 18-08-2021 | 24-06-2022 | Bristol-Myers Squibb Pharma EEIG | yes | yes | yes | no | yes | no |
| Amvuttra | vutrisiran | Treatment of hereditary transthyretin-mediated amyloidosis (hATTR amyloidosis) in adult patients with stage 1 or stage 2 polyneuropathy. | 15-09-2022 | 16-09-2022 | Alnylam Netherlands B.V. | yes | no | no | no | no | no |
| Artesunate Amivas | artesunate | The initial treatment of severe malaria in adults and children. Consideration should be given to official guidance on the appropriate use of antimalarial agents. | 22-11-2021 | 29-04-2022 | Amivas Ireland Ltd | yes | no | no | no | no | no |
| Aspaveli | pegcetacoplan | Treatment of adult patients with paroxysmal nocturnal haemoglobinuria (PNH) who are anaemic after treatment with a C5 inhibitor for at least 3 months. | 13-12-2021 | 24-02-2022 | Swedish Orphan Biovitrum AB (publ) | yes | no | no | no | no | no |
| Breyanzi | lisocabtagene maraleucel | Treatment of adult patients with diffuse large B-cell lymphoma (DLBCL), high grade B-cell lymphoma (HGBCL), primary mediastinal large B-cell lymphoma (PMBCL) and follicular lymphoma grade 3B (FL3B), after two or more lines of systemic therapy. | 4-04-2022 | 26-10-223 | Bristol-Myers Squibb Pharma EEIG | no | yes | no | no | yes | yes |
| Brukinsa | zanubrutinib | Monotherapy for the treatment of adult patients with Waldenström’s macroglobulinaemia (WM) who have received at least one prior therapy, or in first line treatment for patients unsuitable for chemo-immunotherapy. | 22-11-2021 | 06-01-2023 | BeiGene Ireland Ltd | no | no | no | no | no | no |
| Bylvay | odevixibat | Treatment of progressive familial intrahepatic cholestasis (PFIC) in patients aged 6 months or older. | 16-07-2021 | 07-09-2021 | Albireo AB | yes | yes | no | yes | yes | no |
| Carvykti | ciltacabtagene autoleucel | Treatment of adult patients with relapsed and refractory multiple myeloma, who have received at least three prior therapies, including an immunomodulatory agent, a proteasome inhibitor and an anti-CD38 antibody, and have demonstrated disease progression on the last therapy. | 25-05-2022 | 04-01-2023 | Janssen-Cilag International NV | yes | yes | yes | no | yes | no |
| Copiktra | duvelisib | Monotherapy for the treatment of adult patients with:  Relapsed or refractory chronic lymphocytic leukaemia (CLL) after at least two prior therapies. Follicular lymphoma (FL) that is refractory to at least two prior systemic therapies. | 19-05-2021 | 26-05-2021 | Verastem Europe GmbH | no | no | no | no | no | no |
| Ebvallo | tabelecleucel | Monotherapy for treatment of adult and paediatric patients 2 years of age and older with relapsed or refractory Epstein-Barr virus positive post-transplant lymphoproliferative disease (EBV+ PTLD) who have received at least one prior therapy. For solid organ transplant patients, prior therapy includes chemotherapy unless chemotherapy is inappropriate. | 16-12-2022 | 22-05-2023 | Atara Biotherapeutics Ireland Limited | yes | yes | no | yes | yes | yes |
| Elzonris | tagraxofusp | Monotherapy for the first-line treatment of adult patients with blastic plasmacytoid dendritic cell neoplasm (BPDCN). | 7-01-2021 | 15-10-2021 | Stemline Therapeutics B.V. | yes | yes | no | yes | no | no |
| Enhertu | trastuzumab deruxtecan | Monotherapy is indicated for the treatment of adult patients with unresectable or metastatic her2-positive breast cancer who have received two or more prior anti-her2-based regimens. | 18-01-2021 | 16-10-2024 | Daiichi Sankyo Europe GmbH | no | yes | yes | no | no | no |
| Enjaymo | sutimlimab | Treatment of haemolytic anaemia in adult patients with cold agglutinin disease (CAD). | 15-11-2022 | N/A | Genzyme Europe B.V. | yes | no | no | no | no | no |
| Enspryng | satralizumab | Monotherapy or in combination with immunosuppressive therapy (IST) for the treatment of neuromyelitis optica spectrum disorders (NMOSD) in adult and adolescent patients from 12 years of age who are anti-aquaporin-4 IgG (AQP4-IgG) seropositive. | 24-06-2021 | 21-11-2023 | Roche Registration GmbH | yes | yes | no | no | no | no |
| Evrysdi | risdiplam | Treatment of 5q spinal muscular atrophy (SMA) in patients 2 months of age and older, with a clinical diagnosis of SMA Type 1, Type 2 or Type 3 or with one to four SMN2 copies. | 26-03-2021 | 20-05-2021 | Roche Registration GmbH | yes | yes | no | no | yes | yes |
| Filsuvez | birch bark extract | Treatment of partial thickness wounds associated with dystrophic and junctional epidermolysis bullosa (EB) in patients 6 months and older. | 21-06-2022 | 11-08-2022 | Amryt Pharmaceuticals DAC | yes | no | no | no | no | no |
| Gavreto | pralsetinib | Monotherapy for the treatment of adult patients with rearranged during transfection (RET) fusion-positive advanced non-small cell lung cancer (NSCLC) not previously treated with a RET inhibitor. | 18-11-2021 | 14-01-2022 | Roche Registration GmbH | no | no | yes | no |  | no |
| Imcivree | setmelanotide | Treatment of obesity and the control of hunger associated with genetically confirmed Bardet Biedl syndrome (BBS), loss-of-function biallelic pro-opiomelanocortin (POMC), including PCSK1, deficiency or biallelic leptin receptor (LEPR) deficiency in adults and children 6 years of age and above. | 16-07-2021 | 15-09-2021 | Rhythm Pharmaceuticals Limited | yes | yes | no | no | yes | no |
| Inrebic | fedratinib | Treatment of disease-related splenomegaly or symptoms in adult patients with primary myelofibrosis, post polycythaemia vera myelofibrosis or post essential thrombocythaemia myelofibrosis who are Janus Associated Kinase (JAK) inhibitor naïve or have been treated with ruxolitinib. | 8-02-2021 | 16-04-2021 | Celgene Europe B.V. | yes | no | no | no | no | no |
| Jemperli | dostarlimab | Monotherapy of adult patients with mismatch repair deficient (dMMR)/microsatellite instability-high (MSI H) recurrent or advanced endometrial cancer (EC) that has progressed on or following prior treatment with a platinum-containing regimen. | 21-04-2021 | 04-06-2021 | GlaxoSmithKline (Ireland) Limited | no | yes | yes | no | no | yes |
| Kimmtrak | tebentafusp | Monotherapy for the treatment of human leukocyte antigen (HLA)-A*02:01-positive adult patients with unresectable or metastatic uveal melanoma. | 1-04-2022 | 07-06-2022 | Immunocore Ireland Limited | yes | yes | no | no | no | yes |
| Koselugo | selumetinib | Treatment of symptomatic, inoperable plexiform neurofibromas (PN) in paediatric patients with neurofibromatosis type 1 (NF1) aged 3 years and above | 17-06-2021 | 9-08-2021 | AstraZeneca AB | yes | no | yes | no | no | no |
| Livmarli | Maralixibat chloride | Treatment of cholestatic pruritus in patients with Alagille syndrome (ALGS) 2 months of age and older. | 9-12-2022 | 10-02-2023 | Takeda Pharmaceuticals International AG Ireland Branch | yes | no | no | yes | no | yes |
| Livtencity | maribavir | Treatment of cytomegalovirus (CMV) infection and/or disease that are refractory (with or without resistance) to one or more prior therapies, including ganciclovir, valganciclovir, cidofovir or foscarnet in adult patients who have undergone a haematopoietic stem cell transplant (HSCT) or solid organ transplant (SOT). Consideration should be given to official guidance on the appropriate use of antiviral agents. | 9-11-2022 | 11-11-2022 | Takeda Pharmaceuticals International AG Ireland Branch | yes | no | no | no | no | no |
| Lumoxiti | moxetumomab pasudotox | Monotherapy for the treatment of adult patients with relapsed or refractory hairy cell leukaemia (HCL) after receiving at least two prior systemic therapies, including treatment with a purine nucleoside analogue (PNA). | 8-02-2021 | N/A | AstraZeneca AB | yes | no | no | yes | no | no |
| Lumykras | sotorasib | Treatment of adults with advanced non-small cell lung cancer (NSCLC) with KRAS G12C mutation and who have progressed after at least one prior line of systemic therapy. | 6-01-2022 | 8-09-2021 | Amgen Europe BV | no | no | yes | no | no | no |
| Lunsumio | mosunetuzumab | Treatment of adult patients with relapsed or refractory follicular lymphoma (FL) who have received at least two prior systemic therapies. | 3-06-2022 | 4-10-2022 | Roche Registration GmbH | yes | yes | yes | no | no | yes |
| Minjuvi | tafasitamab | In combination with lenalidomide followed by Minjuvi monotherapy for the treatment of adults with relapsed or refractory diffuse large B-cell lymphoma (DLBCL) who are not eligible for autologous stem cell transplant (ASCT)., | 26-08-2021 | 8-10-2021 | Incyte Biosciences Distribution B.V. | yes | no | yes | no | no | no |
| Nexpovio | selinexor | In combination with dexamethasone for the treatment of multiple myeloma in adult patients who have received at least four prior therapies and whose disease is refractory to at least two proteasome inhibitors, two immunomodulatory agents and an anti-CD38 monoclonal antibody, and who have demonstrated disease progression on the last therapy. | 26-03-2021 | 26-05-2021 | Karyopharm Europe GmbH | no | yes | yes | no | no | no |
| Ngenla | somatrogon | treatment of children and adolescents from 3 years of age with growth disturbance due to insufficient secretion of growth hormone. | 14-02-2022 | 25-03-2022 | Pfizer Europe MA EEIG | yes | no | no | no | no | no |
| Nulibry | fosdenopterin | Treatment of patients with molybdenum cofactor deficiency (MoCD) Type A. | 15-09-2022 | 9-04-2024 | Comharsa Life Sciences Limited | yes | yes | no | yes | no | no |
| Onureg | azacitidine | Maintenance therapy in adult patients with acute myeloid leukaemia (AML) who achieved complete remission (CR) or complete remission with incomplete blood count recovery (CRi) following induction therapy with or without consolidation treatment and who are not candidates for, including those who choose not to proceed to, hematopoietic stem cell transplantation (HSCT). | 17-06-2021 | 1-07-2021 | Bristol-Myers Squibb Pharma EEIG | no | no | no | no | no | yes |
| Opdualag | relatlimab / nivolumab | First-line treatment of advanced (unresectable or metastatic) melanoma in adults and adolescents 12 years of age and older with tumour cell pd-l1 expression < 1%. | 15-09-2022 | 27-12-2023 | Bristol Myers Squibb Pharma EEIG | no | no | no | no | no | no |
| Oxbryta | Voxelotor | Treatment of haemolytic anaemia due to sickle cell disease (SCD) in adults and paediatric patients 12 years of age and older as monotherapy or in combination with hydroxycarbamide. | 14-02-2022 | 25-07-2022 | Global Blood Therapeutics Netherlands B.V. | yes | no | no | no | yes | yes |
| Padcev | enfortumab vedotin | Monotherapy for the treatment of adult patients with locally advanced or metastatic urothelial cancer who have previously received a platinum-containing chemotherapy and a programmed death receptor 1 or programmed death ligand 1 inhibitor. | 13-04-2022 | 22-04-2022 | Astellas Pharma Europe B.V. | no | yes | no | no | no | yes |
| Pemazyre | pemigatinib | Monotherapy for the treatment of adults with locally advanced or metastatic cholangiocarcinoma with a fibroblast growth factor receptor 2 (FGFR2) fusion or rearrangement that have progressed  after at least one prior line of systemic therapy. | 26-03-2021 | 7-04-2021 | Incyte Biosciences Distribution B.V. | yes | no | yes | no | no | yes |
| Pepaxti | melphalan flufenamide | In combination with dexamethasone, for the treatment of adult patients with multiple myeloma who have received at least three prior lines of therapies, whose disease is refractory to at least one proteasome inhibitor, one immunomodulatory agent, and one anti-CD38 monoclonal antibody, and who have demonstrated disease progression on or after the last therapy.  For patients with a prior autologous stem cell transplantation, the time to progression should be at least 3 years from transplantation. | 17-08-2022 | 11-11-2022 | Oncopeptides AB (publ) | no | no | no | yes | no | yes |
| Pyrukynd | mitapivat | Treatment of pyruvate kinase deficiency (PK deficiency) in adult patients. | 9-11-2022 | 20-12-2022 | Agios Netherlands B.V. | yes | no | no | no | no | no |
| Qinlock | ripretinib | Treatment of adult patients with advanced gastrointestinal stromal tumour (GIST) who have received prior treatment with three or more kinase inhibitors, including imatinib. | 18-11-2021 | 20-12-2021 | Deciphera Pharmaceuticals (Netherlands) B.V. | yes | no | no | no | no | no |
| Retsevmo | selpercatinib | Monotherapy is indicated for the treatment of adults with:   advanced ret fusion-positive non-small cell lung cancer (NSCLC) who require systemic therapy following prior treatment with immunotherapy and/or platinum-based chemotherapy   advanced RET fusion-positive thyroid cancer who require systemic therapy following prior treatment with sorafenib and/or lenvatinib  Monotherapy is indicated for the treatment of adults and adolescents 12 years and older with advanced RET-mutant medullary thyroid cancer (MTC) who require systemic therapy following prior treatment with cabozantinib and/or vandetanib. | 11-02-2021 | 26-02-2021 | Eli Lilly Nederland B.V. | no | no | yes | no | no | yes |
| Roctavian | Valoctocogene roxaparvovec | Treatment of severe haemophilia A (congenital factor VIII deficiency) in adult patients without a history of factor VIII inhibitors and without detectable antibodies to adeno-associated virus serotype 5 (AAV5)., | 24-08-2022 | N/A | BioMarin International Limited | yes | yes | yes | no | yes | no |
| Rybrevant | amivantamab | Monotherapy for treatment of adult patients with locally advanced or metastatic non-small cell lung cancer (NSCLC) with activating epidermal growth factor receptor (EGFR) Exon 20 insertion mutations, after failure of platinum-based chemotherapy. | 9-12-2021 | 15-07-2024 | Janssen-Cilag International N.V. | no | no | yes | no | no | yes |
| Scemblix | asciminib | Treatment of adult patients with Philadelphia chromosome positive chronic myeloid leukaemia in chronic phase (Ph+ CML CP) previously treated with two or more tyrosine kinase inhibitors. | 25-08-2022 | 15-06-2022 | Novartis Europharm Limited | yes | no | no | no | no | yes |
| Skysona | elivaldogene autotemcel | Treatment of early cerebral adrenoleukodystrophy in patients less than 18 years of age, with an abcd1 genetic mutation, and for whom a human leukocyte antigen (hla)-matched sibling haematopoietic stem cell (hsc) donor is not available | 16-7-2021 | N/A | bluebird bio (Netherlands) B.V. | yes | yes | yes | yes | yes | no |
| Skytrofa (previously Lonapegsomatropin Ascendis Pharma) | lonapegsomatropin | Treatment of early cerebral adrenoleukodystrophy in patients less than 18 years of age, with an abcd1 genetic mutation, and for whom a human leukocyte antigen (hla)-matched sibling haematopoietic stem cell (hsc) donor is not available | 11-01-2022 | 17-10-2022 | Ascendis Pharma Endocrinology Division A/S | yes | no | no | no | no | no |
| Sogroya | somapacitan | Replacement of endogenous growth hormone (GH) in adults with growth hormone deficiency (AGHD). | 31-03-2021 | 22-10-2021 | Novo Nordisk A/S | =yes | no | no | no | no | no |
| Tabrecta | capmatinib | Monotherapy for the treatment of adult patients with advanced non-small cell lung cancer (NSCLC) harbouring alterations leading to mesenchymal epithelial transition factor gene exon 14 (METex14) skipping, who require systemic therapy following prior treatment with immunotherapy and/or platinum based chemotherapy. | 20-06-2022 | 27-03-2023 | Novartis Europharm Limited | no | no | no | no | no | yes |
| Tavneos | avacopan | In combination with a rituximab or cyclophosphamide regimen for the treatment of adult patients with severe, active granulomatosis with polyangiitis (GPA) or microscopic polyangiitis (MPA). | 11-01-2022 | 6-05-2022 | Vifor Fresenius Medical Care Renal Pharma France | yes | no | no | no | yes | yes |
| Tecvayli | teclistamab | Monotherapy for the treatment of adult patients with relapsed and refractory multiple myeloma, who have received at least three prior therapies, including an immunomodulatory agent, a proteasome inhibitor, and an anti-CD38 antibody and have demonstrated disease progression on the last therapy. | 23-08-2022 | 9-11-2022 | Janssen-Cilag International N.V. | no | yes | yes | no | yes | yes |
| Tepmetko | tepotinib | Monotherapy for the treatment of adult patients with advanced non-small cell lung cancer (NSCLC) harbouring alterations leading to mesenchymal-epithelial transition factor gene exon 14 (METex14) skipping, who require systemic therapy following prior treatment with immunotherapy and/or platinum-based chemotherapy. | 16-02-2022 | 24-09-2021 | Merck Europe B.V. | no | no | no | no | no | yes |
| Trodelvy | sacituzumab govitecan | Monotherapy for the treatment of adult patients with unresectable or metastatic triple-negative breast cancer (mTNBC) who have received two or more prior systemic therapies, including at least one of them for advanced disease. | 22-11-2021 | 8-09-2021 | Gilead Sciences Ireland UC | no | yes | no | no | no | yes |
| Tukysa | tucatinib | In combination with trastuzumab and capecitabine for the treatment of adult patients with HER2‑positive locally advanced or metastatic breast cancer who have received at least 2 prior anti‑HER2 treatment regimens. | 11-02-2021 | 19-02-2021 | Seagen B.V. | no | no | no | no | no | no |
| Upstaza | eladocagene exuparvovec | Treatment of patients aged 18 months and older with a clinical, molecular, and genetically confirmed diagnosis of aromatic L amino acid decarboxylase (AADC) deficiency with a severe phenotype (see section 5.1). | 18-07-2022 | 17-11-2022 | PTC Therapeutics International Limited | yes | no | no | yes | no | no |
| Voraxaze | glucarpidase | To reduce toxic plasma methotrexate concentration in adults and children (aged 28 days and older) with delayed methotrexate elimination or at risk of methotrexate toxicity. | 11-01-2022 | 15-06-2023 | SERB SAS | yes | no | no | yes | no | no |
| Voxzogo | vosoritide | Treatment of achondroplasia in patients 2 years of age and older whose epiphyses are not closed. The diagnosis of achondroplasia should be confirmed by appropriate genetic testing. | 26-08-2021 | N/A | BioMarin International Limited | yes | no | no | no | no | no |
| Vyvgart | efgartigimod alfa | Add-on to standard therapy for the treatment of adult patients with generalised myasthenia gravis (gMG) who are anti acetylcholine receptor (AChR) antibody positive. | 10-08-2022 | 14-03-2024 | Argenx BV | yes | no | no | no | no | yes |
| Xenpozyme | olipudase alfa | Enzyme replacement therapy for the treatment of non-Central Nervous System (CNS) manifestations of Acid Sphingomyelinase Deficiency (ASMD) in paediatric and adult patients with type A/B or type B. | 24-06-2022 | 1-08-2022 | Genzyme Europe B.V. | yes | yes | no | no | yes | yes |
| Zokinvy | lonafarnib | Treatment of patients 12 months of age and older with a genetically confirmed diagnosis of Hutchinson-Gilford progeria syndrome or a processing-deficient progeroid laminopathy associated with either a heterozygous LMNA mutation with progerin-like protein accumulation or a homozygous or compound heterozygous ZMPSTE24 mutation. | 18-07-2022 | 24-08-2022 | EigerBio Europe Limited | yes | yes | no | yes | no | no |
| Zynlonta | loncastuximab tesirine | Monotherapy for the treatment of adult patients with relapsed or refractory diffuse large B-cell lymphoma (DLBCL) and high-grade B-cell lymphoma (HGBL), after two or more lines of systemic therapy. | 20-12-2022 | 7-02-2023 | ADC Therapeutics (NL) B.V. | no | no | yes | no | no | no |

**Supplementary Table S2**. Data sources regarding CUPs for anticancer medicines and orphan medicines centrally authorized in 2021 and 2022. EMA: European Medicines Agency. EMRD: European Medicines Regulatory Database. EPAR: European Public Assessment Report. EU: European Union. *See Supplement 3 for complete overview of national webpages in the 7 European countries.

|  | Tradename | Active substance name | Indication | Date of marketing authorization in the EU | Marketing authorization holder | Orphan designation | Anticancer medicine | Accelerated assessment | Conditional approval | Exceptional circumstances | PRIME | Compassionate use program start and end date | Compassionate use indication | Reimbursement of compassionate use |
| --- | --- | --- | --- | --- | --- | --- | --- | --- | --- | --- | --- | --- | --- | --- |
| EMA EPAR database | x | x | x | x | x | x | x | x | x | x |  |  |  |  |
| EMRD | x | x | x | x | x | x | x | x | x | x | x |  |  |  |
| National competent authority websites* |  |  |  |  |  |  |  |  |  |  |  | x | x |  |
| National reimbursement organization websites* |  |  |  |  |  |  |  |  |  |  |  |  |  | x |

**Supplementary Table S3.** Overview of governmental webpages used to retrieve data on Compassionate Use Programs.

| **Country** | **Webpage name** | **URL** |
| --- | --- | --- |
| Belgium | Gebruik in schrijnende gevallen - medische noodprogramma's | https://www.fagg-afmps.be/nl/MENSELIJK_gebruik/geneesmiddelen/geneesmiddelen/onderzoek_ontwikkeling/gebruik_in_schrijnende_gevallen_medische_noodprogrammas |
|  | Geschorste programma’s | https://www.fagg.be/nl/MENSELIJK_gebruik/geneesmiddelen/geneesmiddelen/onderzoek_ontwikkeling/gebruik_in_schrijnende_5 |
|  | Gesloten programma’s | https://www.fagg.be/nl/MENSELIJK_gebruik/geneesmiddelen/geneesmiddelen/onderzoek_ontwikkeling/gebruik_in_schrijnende_4 |
|  | Onbeantwoorde medische behoeften - Unmet Medical Need | https://www.inami.fgov.be/nl/thema-s/verzorging-kosten-en-terugbetaling/wat-het-ziekenfonds-terugbetaalt/geneesmiddelen/geneesmiddel-terugbetalen/onbeantwoorde-medische-behoeften-unmet-medical-need |
|  | Vergoedbare geneesmiddelen en radio-farmaceutische producten | https://webappsa.riziv-inami.fgov.be/ssp/ProductSearch |
| France | Recherche advance | https://www.has-sante.fr/jcms/fc_2874928/fr/recherche-avancee |
| Germany | Aktuell laufende und bestätigte Arzneimittel-Härtefallprogramme | https://www.bfarm.de/DE/Arzneimittel/Klinische-Pruefung/Compassionate-Use/compUse-tabelle.html;jsessionid=97D6BAA72A47916B0536EE2F062220B7.intranet232?nn=701486 |
| Netherlands | Overzicht van goedgekeurde compassionate use programma's | https://www.cbg-meb.nl/onderwerpen/hv-compassionate-use-programma/overzicht-goedgekeurde-cup |
| Norway | Compassionate Use Program (CUP) Approved by The Norwegian Medicinal Products Agency | https://www.dmp.no/contentassets/b71d6f2f34264dd7a22822deab47c201/compassionate-use-program_eng_2024-01-12.pdf |
| Switzerland | Swiss Medic Search page | https://www.swissmedic.ch/swissmedic/en/home/suche.html#Temporary%20authorisation%20 |
| United Kingdom | Early access to medicines scheme (EAMS): scientific opinions | https://www.gov.uk/government/collections/early-access-to-medicines-scheme-eams-scientific-opinions |
|  | Expired early access to medicines scheme scientific opinions | https://www.gov.uk/government/publications/early-access-to-medicines-scheme-expired-scientific-opinions/expired-early-access-to-medicines-scheme-scientific-opinions |

**Supplementary Figure S1**. Timeline for pre-authorization access routes before reimbursement. Some countries allow compassionate use programs until marketing authorization, others until reimbursement. EMA: European Medicines Agency. EU: European Union. HTA: Health Technology Assessment.

**
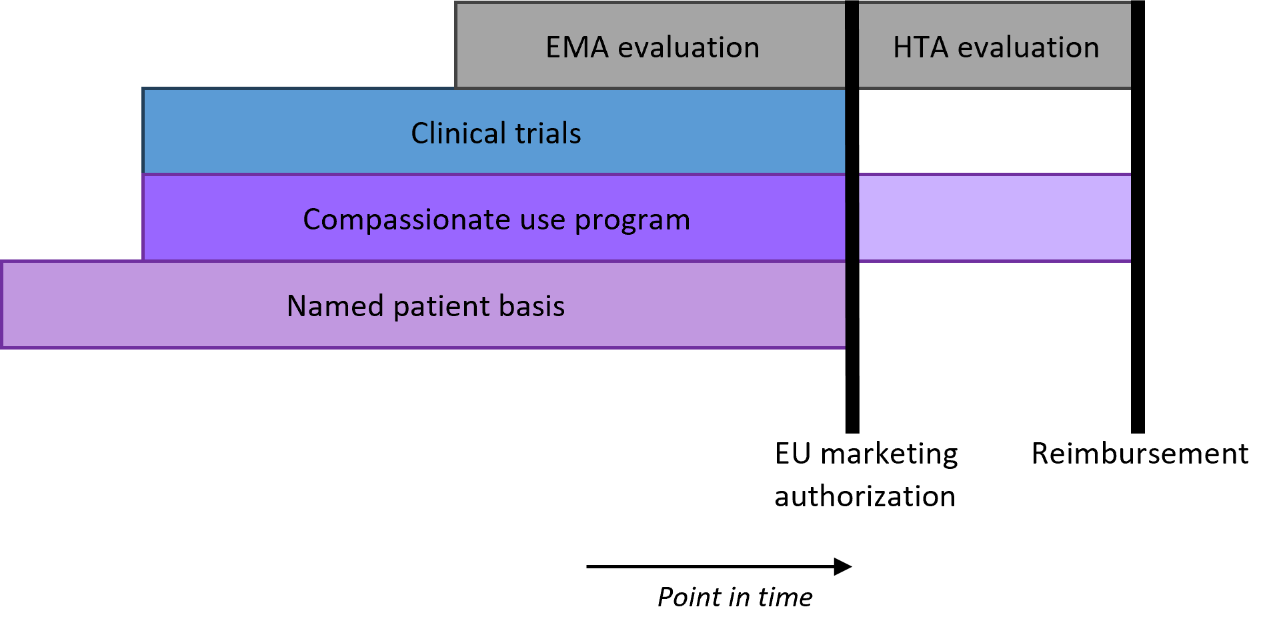
**

**Supplementary Figure S2**. Flow diagram of sample selection of anticancer and orphan medicines authorized in 2021 and 2022. Data was collected from the European Medicines Agency’s European Public Assessment Report (EPAR) database and supplemented with data from the European Medicines Regulatory Database (EMRD). Green boxes represent inclusion, while red boxes illustrate exclusion. Medicines with one of the following legal bases were excluded: generic (article 10(1)), hybrid article (10(3)), biosimilar (article 10(4)), well-established use (article 10a), or informed consent (article 10c). Only originator medicines were included (article 8(3)). Anticancer medicines were included by selecting medicines from the therapeutic subgroup “ATC code L01 Antineoplastic agents”. Orphan medicines were included by selecting medicines that had an orphan designation at the time of the granting of the marketing authorization. Other medicines were excluded. EU: European Union.


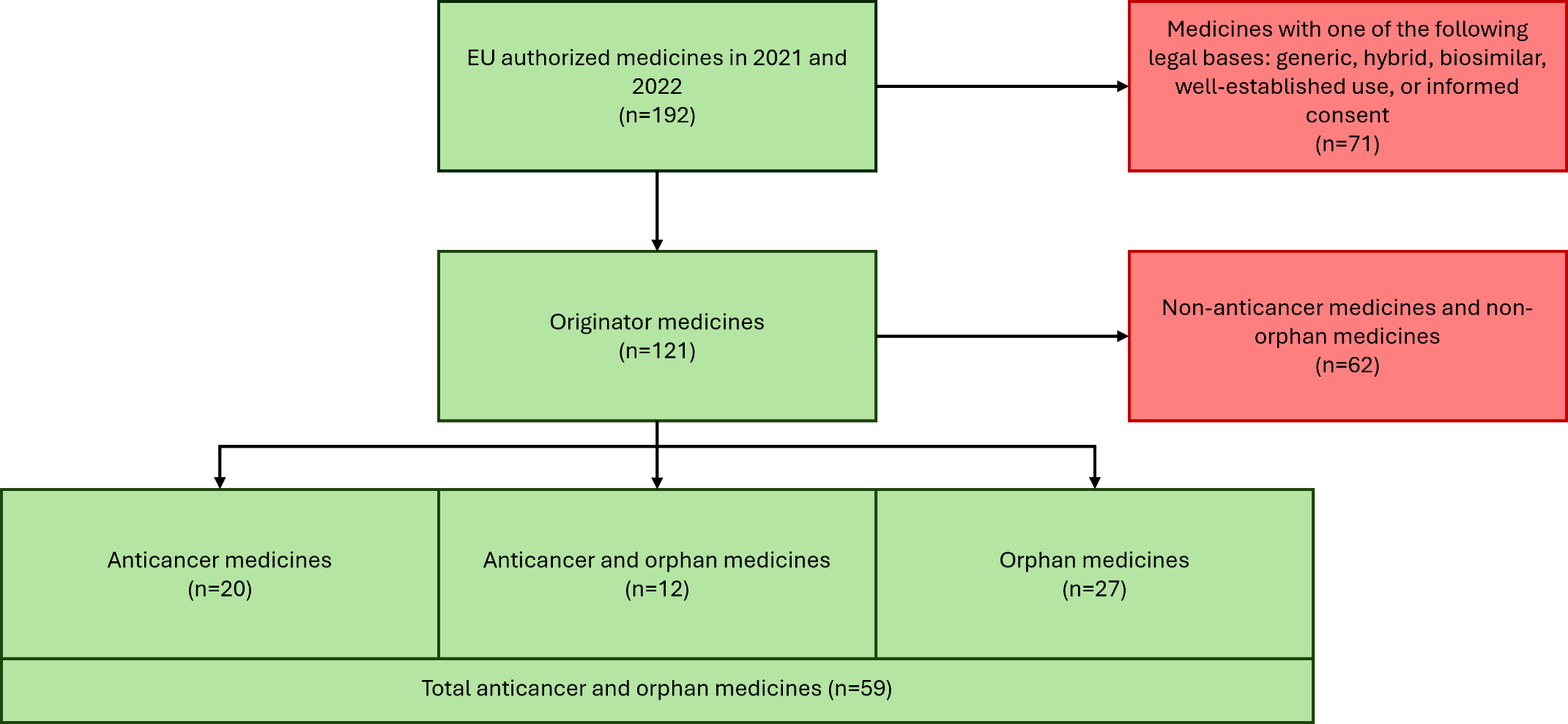

Supplement: Supplementary Material [file mmc1.docx]
